# COSMIC Cancer Gene Census 3D database: understanding the impacts of mutations on cancer targets

**DOI:** 10.1093/bib/bbab220

**Published:** 2021-06-17

**Authors:** Ali F Alsulami, Pedro H M Torres, Ismail Moghul, Sheikh Mohammed Arif, Amanda K Chaplin, Sundeep Chaitanya Vedithi, Tom L Blundell

**Affiliations:** Department of Biochemistry at the University of Cambridge, Cambridge CB2 1GA, UK; Laboratório de Modelagem e Dinâmica Molecular, Instituto de Biofísica Carlos Chagas Filho, Universidade Federal do Rio de Janeiro, Rio de Janeiro, RJ, Brasil; UCL Cancer Institute, University College London, UK; Department of Biochemistry, University of Cambridge, Cambridge CB2 1GA, UK; Department of Biochemistry, University of Cambridge, Cambridge CB2 1GA, UK; Department of Biochemistry, University of Cambridge, Cambridge CB2 1GA, UK; Department of Biochemistry, University of Cambridge, Cambridge CB2 1GA, UK

**Keywords:** Cancer Gene Census 3D, hallmark mutations, modelling cancer genes census, mutational analyses of cancer drug targets

## Abstract

Mutations in hallmark genes are believed to be the main drivers of cancer progression. These mutations are reported in the Catalogue of Somatic Mutations in Cancer (COSMIC). Structural appreciation of where these mutations appear, in protein–protein interfaces, active sites or deoxyribonucleic acid (DNA) interfaces, and predicting the impacts of these mutations using a variety of computational tools are crucial for successful drug discovery and development. Currently, there are 723 genes presented in the COSMIC Cancer Gene Census. Due to the complexity of the gene products, structures of only 87 genes have been solved experimentally with structural coverage between 90% and 100%. Here, we present a comprehensive, user-friendly, web interface (https://cancer-3d.com/) of 714 modelled cancer-related genes, including homo-oligomers, hetero-oligomers, transmembrane proteins and complexes with DNA, ribonucleic acid, ligands and co-factors. Using SDM and mCSM software, we have predicted the impacts of reported mutations on protein stability, protein–protein interfaces affinity and protein–nucleic acid complexes affinity. Furthermore, we also predicted intrinsically disordered regions using DISOPRED3.

## Introduction

Cancer is a heterogeneous group of diseases, which is caused by the accumulation of mutations in genes that control cell activities, such as proliferation and apoptosis [1]. Mutations reported by the Cancer Genome Consortium (ICGC) [2] and the Catalogue of Somatic Mutations in Cancer (COSMIC) [3] show that most cancer cells possess 60 or more mutations [4]. Of these, 80% are somatic, 10% are germline and 10% are both somatic and germline mutations [5]. Although the majority are neutral passenger mutations, a few are considered to be detrimental or driver mutations [6].

Nowadays, rapid next-generation sequencing technology can detect cancer somatic mutations very quickly, providing essential information concerning which genes have been highly mutated [7]. Non-synonymous single nucleotide polymorphisms (nsSNPs) result in amino acid substitutions that could positively or negatively modulate the protein’s function, and these are often associated with human disease [8]. The standard way to identify candidate driver nsSNP mutations is through frequency-based approaches, which need a large sample size, for example, the frequently observed V600E mutation in BRAF [9], but this approach also presents the drawback of not detecting infrequent driver mutations. An alternative way forward is the functional approach, which detects mutations in conserved regions, also known as orthostatic drivers; this method is used to detect infrequently mutated genes [10]. However, not all mutations in conserved regions are drivers, and conversely, not all mutations in non-conserved regions are passengers [11].

Most mutations in genes vary among different samples of the same cancer type and also vary within the same gene in different cancers [12]. Therefore, identifying driver mutations that lead to a particular type of cancer, to cell growth and proliferation or to drug resistance remains a major challenge. However, there are multiple databases and pipelines describing cancer-driver genes, such as DriverDBv3 [13], a cancer driver gene database, IntOGen [14], which identifies cancer drivers across tumour types and the aforementioned COSMIC and ICGC.

In addition, algorithms such as SIFT [15] and PolyPhen-2 [16] which depend on the analysis of evolutionary substitutions in homologous sequences give clues about the essentiality of individual amino acids for the identification and characterization of putative driver mutations. Furthermore, knowledge of protein three-dimensional (3D) structure allows further understanding of the impacts of mutations on its structure and function. This is exploited in a number of approaches, including SDM [17], which has been developed in our group to predict impacts of mutations on structural stability based on statistical analysis of mutations as a function of the local structure. More recently, the mCSM family of computer programmes [18] has also been developed in our group to exploit the structural knowledge using a machine learning method. This approach depends on a learning set of 3D structures to predict impacts of mutations on the stability of protomers (mCSM-stability) as well as on protein–protein interactions (mCSM-PPI), protein–nucleic acid interactions (mCSM-NA) and protein–ligand interactions (mCSM-lig) [19], among the others. However, although it is clear that the 3D structure can provide useful insights into identifying and understanding driver mutations, experimental definition of the 3D structure, especially for complex multidomain structures and multiprotein assemblies, remains a challenge [11].

Although the number of experimental protein structures deposited in the Protein Data Bank (PDB) [20], as well as the model structures deposited in Genome3D [21] and Model Portal (PMP) [22], has been steadily increasing over the past few decades, few complete experimental structures of multidomain, oligomeric and multicomponent systems have been determined for the human proteome. Of the 723 genes present in the COSMIC Cancer Gene Census (COSMIC CGC) [23], 245 have no hits in the PDB, and of the remaining 478 genes, 263 have less than 50% coverage. Although multiple resources map mutations to the 3D structures, these are largely limited to experimentally solved structures [24]. Comparative modelling based on homologues with relatively high sequence identity (preferably over 30%) is helpful to predict the structures of protomers or individual domains, partially bridging the gap between sequence and structural information. Useful software includes Modeller [25], Phyre2 [26], LOMETS2 [27], DomSerf 2.1 [28] and I-TASSER [29]. More recently, machine learning/artificial intelligence methods, such as AlphaFold [30], have made impressive progressive progress in modelling proteins. However, predicting structures comprising multiple domains that are often connected by flexible regions or higher order multicomponent assemblies remains an even greater challenge.

Although driver mutations are often distributed across the protein sequence, they tend to cluster together in the 3D structure and appear mainly in the active site or at the interface [11], but they can occasionally act allosterically within a protomer or domain, or between protomers in higher order assemblies. In order to understand the ways that mutations can impact on function, we describe the construction of the COSMIC CGC 3D (CGC 3D) database that includes 3D structures for multidomain, homo-oligomeric and multicomponent systems of the human proteome along with the use of 3D structures in the prediction of the impacts of mutations, allowing the identification of rare driver mutations. These data can be useful in guiding drug discovery by identifying regions in the protein which are highly impacted by mutations and by designing new leads that interact with regions in which mutations leading to resistance are less likely to occur.

## Methods

### Modelling gene products in **CGC**

The protein sequences of the genes are taken from the COSMIC database. DISOPRED3 [31] programme is used to predict the disordered regions of the target sequences and to identify regions likely to be disordered. Structural coverage for each gene product present is calculated, the amino acid sequences are searched against the PDB using FUGUE [32], which recognizes distant homologues using combined information from both sequence and structure, PSI-Basic Local Alignment Search Tool (BLAST) [33], which relies on Position-Specific Scoring Matrix (PSSM) profile–profile alignment, and HHsearch [34], which uses hidden Markov models (HHMs) [35]. We also use other software and databases to inform our modelling including: Pfam [36], a large collection of protein families, represented by multiple sequence alignments and HMMs; InterPro [37], providing functional analysis of proteins by classifying them into families and predicting domains and important sites; Simple Modular Architecture Research Tool (SMART) [38] , a web resource (http://smart.embl.de/) providing simple identification and extensive annotation of protein domains and the exploration of protein domain architectures; CATH [34] and SCOP [39] databases for domain annotations as well as UniProt [40] for transmembrane extracellular and intracellular annotations.

**Figure 1:**
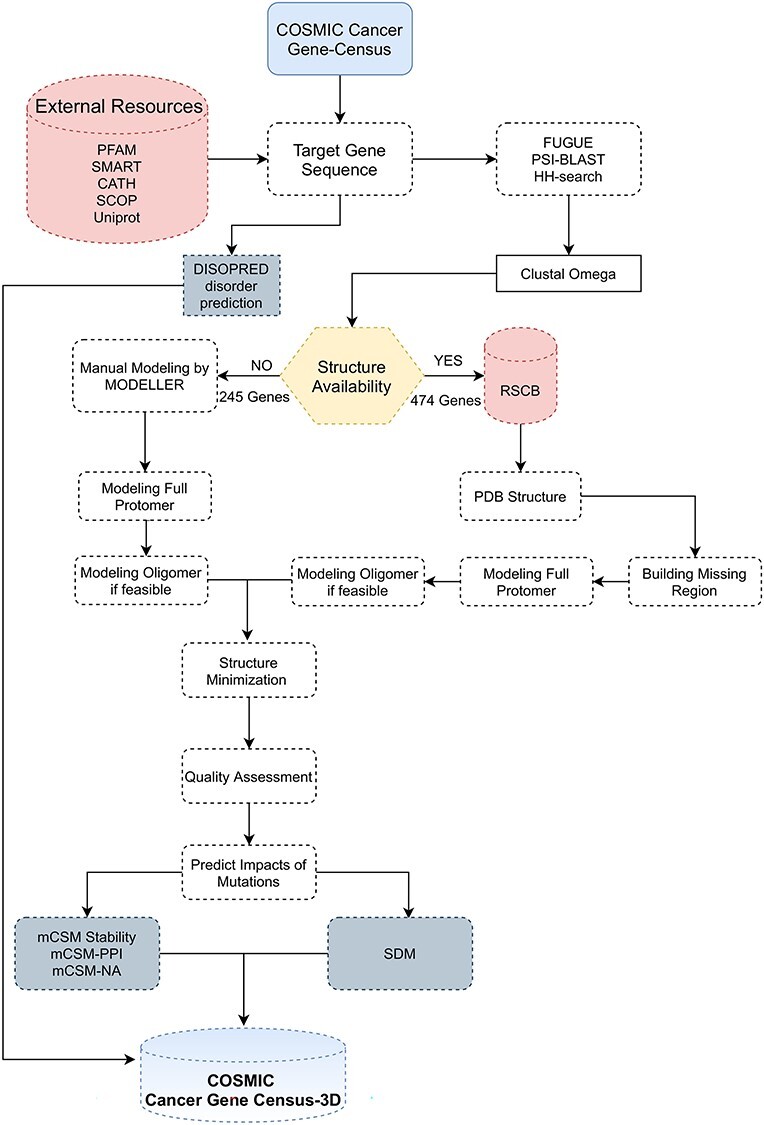
Simplified flowchart for manual modelling pipeline. Starting from target gene in COSMIC CGC, annotating gene domains, finding PDB hits and homologues structures, building model complexes and finally predicting the impact of the mutations on the modelled structures.

All proteins were modelled interactively using the knowledge-based method, MODELLER version 9.23. Where identical domains or larger structures are available, these were associated with the sequences of each of the gene products in the COSMIC CGC. Where no identical experimental structure is identified, homologue regions of known structure were identified by FUGUE, PSI-BLAST, and HHsearch were selected as templates. The selected single or multiple templates from various experimental techniques, such as X-ray, nuclear magnetic resonance (NMR) and Cryo-EM, were based on criteria, such as identity, coverage and resolution. The structures of bitopic transmembrane proteins (also known as single-pass or single-spanning membrane proteins) that have not been solved experimentally were obtained from the Membranome database (OPM) [41], which provides structural and functional information for more than 6000 such proteins from *Homo sapiens*. Issues with selected templates, such as missing atom coordinates for long loops in the PDB files, were taken into consideration when selecting templates in order to avert the building of long loops. Each selected template was re-aligned to the target sequence using Clustal Omega software [42], and the generated sequences alignment file was used by MODELLER to build the final model, which is optimized using side-chain minimization implemented in Foldit [43] to remove residues clashes (Figure 1).

All higher order structures were built based on evidence from the literature and UniProt annotations of the target gene. For gene products with no structural representation in the PDB, the full-length monomers/protomers, and where relevant, the structures of homo- and hetero-oligomers and complexes with deoxyribonucleic acid (DNA), ribonucleic acid (RNA) and small molecule ligands are modelled using the selected templates. For example, for cyclic AMP-dependent transcription factor ATF-1, which has a Basic Leucine Zipper Domain, annotated in UniProt as binding DNA as a dimer, the crystal structure with PDB ID: 5ZKO was selected as a template to produce the final modelled structure (Supplementary Figure S1, see Supplementary Data available online at http://bib.oxfordjournals.org/).

For some gene products, experimental structures are reported in more than one PDB entry. For example, RAC-beta serine/threonine-protein kinase (AKT2) has a pleckstrin homology (PH) domain (PDB ID: 1P6S), covering the region between amino acids 1 and 111, and a protein kinase domain (PDB ID: 3D0E), covering the region between 146 and 480. In this case, the missing residues 112–145, located between the PH domain and kinases domain, are predicted to be disordered. The full protomer structure was obtained by assembling the three regions (PH domain, disordered region and kinase domain), and the homodimer was obtained by re-aligning the modelled protomers to an experimentally solved homodimeric structure (PDB ID: 3D0E) (Supplementary Figure S2, see Supplementary Data available online at http://bib.oxfordjournals.org/).

Genes with experimental structures recorded in separate PDB entries can also exist in different conformations and oligomeric states. For example, the structure of the kinase domain of activin receptor type-1 has been reported in different conformational states (PDB ID: 4DYM, 6GIN) and therefore two models are built. Activin receptor type-1 protein has three regions: the extracellular domain, transmembrane and the cytoplasmic region. The three regions are assembled to obtain the full protomer. This was then superposed on the experimentally solved extracellular hetero 4-mer (PDB ID: 3EVS) and cytoplasmic (PDB ID: 4DYM) to obtain the first hetero-4-mer complexes. To achieve the second reported conformation, the solved protomer was re-aligned to the extracellular hetero 4-mer (PDB ID: 3EVS) and cytoplasmic (PDB ID: 6GIN), as shown in Figure 3**C**.

For all modelled complexes, we implement jsPISA [44] to evaluate the interfaces formed in the protein homo/hetero oligomers and in the protein–DNA structures. Quality assessment for a modelled protein is particularly important when conducting drug design and/or predicting the effects of mutations. Generally speaking, predicting accurate models with Root-mean-square deviation (RMSDs) of less than 1 Å is not possible for large multidomain or multicomponent complexes. Accurate models depend on the availability of templates with high coverage and high-resolution structures, which is often not the case for the genes in the COSMIC CGC. PROCHECK [45] is used as a quality assessment for our modelled structures, with a resolution parameter of 2.4 Å. This tool is very useful to assess the quality of not only globular domains but also intrinsically disordered regions that are integrated into the modelled protomer. PROCHECK outputs include 10 plots that give comprehensive analyses for all the residues. Furthermore, MolProbity [46], another quality assessment tool, is used to evaluate the stereochemical quality, contacts, steric clashes, dihedral angles, H-bonds and side-chain rotamers of the modelled protein structures. The overall MolProbity score is a log-weighted value of all these features.

### Mutation data

All missense mutations are downloaded from the COSMIC database (https://cancer.sanger.ac.uk/cosmic/download). Mutation data are pre-processed and filtered using an in-house Python script and the frequency of each mutant is calculated. The impacts of these mutations, whether they are stabilizing or destabilizing, are predicted from the 3D structure by using two independent software:

(i) SDM: a knowledge-based approach using environment-specific amino acid substitution tables to predict the impacts of mutations on protein stability.
(ii) mCSM: a machine learning approach using a graph-signature method.
  (a) mCSM stability predicts the impacts of mutations on protein stability.
  (b) mCSM-PPI predicts the impacts of mutations at a protein–protein interface.
  (c) mCSM-NA predicts the impacts of mutations on protein–DNA interactions.

#### Web interface

The CGC 3D database has been developed using Express.JS, which is a web application framework for node.js. Data are stored in multiple tables in the PostgreSQL database. Express (version 4) framework is used in the backend to query and retrieve information from the stored tables. The frontend is developed using HTML5, CSS and Bootstrap (version 4). We use Embedded JavaScript (EJS) as a templating engine, which produces the final HTML by injecting data into an HTML template at the client side. All the 3D protein structures can be visualized using MolStar. In addition, the UniProt viewer was implemented to display the sequence, and key annotations were reported in the UniProt database. The MSAViewer [47] was implemented to visualize the multiple sequence alignment. The heatmap was implemented using D3.js library to produce dynamic interactive mutations data.

## Results and database features

CGC 3D has five components:

(i) Search query.
(ii) Gene description and links to other relevant resources.
(iii) Structure visualization and sequence annotations.
(iv) Models and PDB tables.
(v) Mutations table and heatmap.

**Figure 2:**
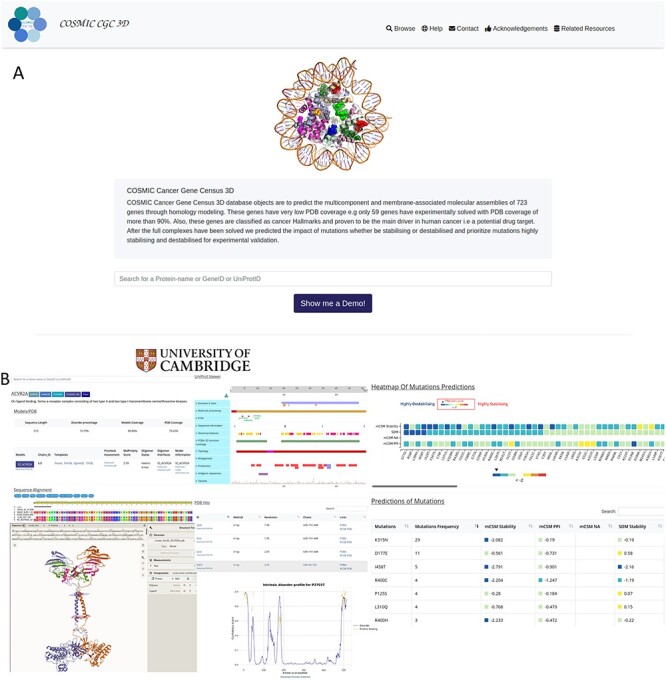
(**A**) Website front and result pages. The ‘navbar’ at the top provides navigation to the Browse, Contact, Help, Acknowledgements and related resources. A brief description of CGC-3D is presented in the midpage and gives the database features followed by a query field. All search queries are performed in one HTML Form via GeneID, Gene Name or UniProtID. (**B**) The Results page includes all data: gene name with links to external data sources, including UniProt, Pfam, COSMIC and COSMIC-3D; a brief description; MolStar which displays 3D structures of the target-gene models; UniProt viewer which is used to visualize domains and other annotations and DISOPRED3 plot, which predicts disorder of the target gene. Models and PDB tables provide information about the modelled and experimental structures, and the heatmap and mutations table provide predictions of the impacts of mutations reported in COSMIC CGC.

From the main CGC 3D entry site, the database can be queried in three ways (gene-id, UniProt-id and gene-name), producing graphical and tabular displays of the data. The Results page includes: UniProt viewer that provides the user with essential biological information; MolStar for visualizing the model/PDB structures; DISOPRED3, which generates a disorder prediction graph and three tables (Figure 2):

(i) The mutations table has each mutant with the predicted values from multiple software: SDM, mCSM stability, mCSM-PPI, mCSM-NA as well as the frequency for each mutant, as reported in COSMIC CGC.
(ii) PDB table provides solved crystal structures, where available.
(iii) Models/PDB table includes modelled protomers, oligomeric complexes and proteins that had their structures solved experimentally with high coverage. The table also includes quality assessments, oligomeric interface analysis and a downloadable text file with further model information. For genes with full structural coverage, the structures with the best resolution are selected, downloaded from the RCSB PDB and saved as biological assemblies and represented in the Models/PDB table. Whereas, other structures are presented in the PDB table.

Each gene has a short description parsed from UniProt, along with external links to other resources, such as COSMIC, COSMIC-3D, UniProt, MobiDB [48] and Pfam, to reduce data redundancy. MolStar, a web-based tool supported by HTML5, is implemented to view the 3D protein structure of 714 gene products. It has multiple features such as a PDB parser, which directly loads the structures from PDB. Furthermore, it shows interactions such as hydrogen bonding, *π*–*π* stacking interactions of selected ligands and amino acid residues, which could assist in visualizing the gain or loss of interactions between the mutant and the wild-type residues.

**Figure 3:**
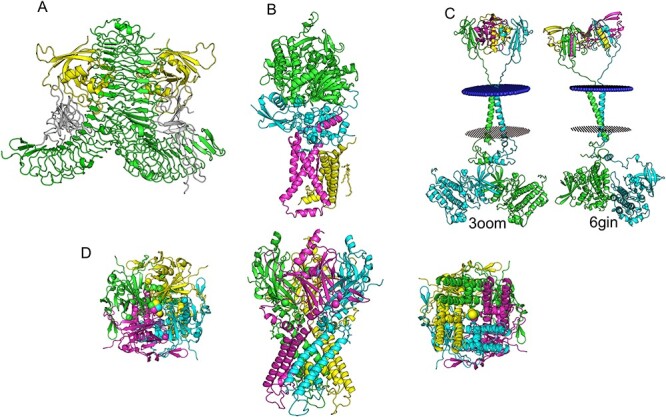
Four modelled oligomeric multicomponent targets selected from the CGC database. (**A**) RSPO3, an enhancer for the WNT signalling, is coloured in white, the E3 ubiquitin–protein ligase RNF43 is coloured in yellow. The LGR5 homodimer is coloured in green. (**B**) Heterotetramer of FAD-binding protein (green), iron–sulphur protein (cyan), SDHC (magenta) and SDHD (yellow). The heme molecule is represented between SDHC and SDHD in stick magenta. (**C**) Activin receptor type-1 (ACVR1) heterotetramer comprising ACVR1 homodimer represented in green and cyan. Ligand in the cytoplasm region is coloured in yellow and magenta, and the transmembrane region is highlighted between red/blue circular structure, which represents the protein membrane region. Two different conformations for the kinase domain are represented, and they were based on PDB entries 3OOM (left) and 6GIN (right). (**D**) The homotetrameric structure of KCNJ5 with each protomer coloured differently. Top and bottom views show the potassium ions inside the channel.

The sequence annotations, visualized using the ProtVista viewer, show domain annotations, post-translational modifications, transmembrane regions, variants and multiple other features. Visualizing all these features in one viewer helps understanding the protein’s role and function.

The Models table contains a quality assessment for each model, a total disorder percentage, the gene-sequence length, the templates selected to build the model and calculated model/PDB coverages.

The three largest models deposited into the COSMIC CGC 3D database are LRP1B (4599 residues), FAT1 (4588 residues) and FAT3 (4557 residues). The smallest gene is COX6C, coding for 75 amino acid residues, whereas the largest reported gene in the CGC is MUC16, coding for 14 507 amino acids. Users can download and view the structures of all the models, including monomers, homo- and hetero- oligomers, together with PROCHECK quality assessment, model information and disorder prediction graph.

To illustrate some of the models that have been built and deposited into the COSMIC CGC 3D databases, we have selected several hetero-oligomeric complexes:

(i) R-spondin 3 (RSPO3) acts as an enhancer for the WNT signalling. It binds to E3 ubiquitin ligase (RNF43) as well as leucine-rich-repeat-containing G-protein-coupled receptor (GPCR) (LGR4-6). This tripartite interaction precludes degradation of the WNT receptor. The structure of this complex hetero 6-mer will give greater insight into RSPO3 function. The templates used to build the RSPO3 hetero 6-mer complex were entries 4C8V and 4KNG. The TM-align and the RMSD between the model and the template are 0.86 Å and 2.64 Å, respectively. The MolProbity score of 3.51 indicates this model is likely to be correct and could be used to assess the impact of mutations using *in silico* methods or for molecular docking to identify small molecules that bind at the protein–protein interface and inhibit WNT receptor degradation (Figure 3**A**).
(ii) The G-protein-activated inward rectifier potassium channel 4 (KCNJ5). This is a voltage-dependent channel, controlled by GPCRs, which allows potassium K^+^ ions to flow into the cell based on the potassium concentration outside the cell. KCNJ5 is modelled as a homo 4-mer based on PDB entry 3SYP; it presents RMSD of 1.72 Å and a TM-score of 0.95 between the template and the model and a MolProbity score of 2.70. The interfaces calculated by jsPISA display a large circular area (Supplementary Figure S4, see Supplementary Data available online at http://bib.oxfordjournals.org/), where the larger the radius, the greater the likelihood of finding interfaces within the biological assemblage. This modelled structure could be used in a molecular dynamics simulation to study the potassium channel mechanism and dynamics, or by molecular docking to look for small ligands interrupting the potassium flow (Figure 3**D**).
(iii) Activin A receptor 1 (ACVR1), known as ALK-2, is a transmembrane receptor, belonging to the transforming growth factor beta (TGF-β) receptor family, which signals through a heteromeric complexes of type I or II. This protein is important for the bone morphogenetic pathway, responsible for skeletal system repair and development. The ACVR1 models include ligands bound to the extracellular cysteine-rich region. The final models are hybrids based on the solved crystal structure of the kinase domain and close homologues of the extracellular region. Multiple templates were selected to build two models with different conformational states. The first modelled structure is based on PDB ID: 3EVS_C, 1BTE_A/B, 4C02_A, 6GIN_A/B, with MolProbity score of 2.98, and the second modelled structure is based on PDB ID: 3EVS_C, 1BTE_A/B, 4C02_A, 3OOM_A, 4DYM_A), with MolProbity score of 3.17 (Figure 3**C**).
(iv) Succinate dehydrogenase cytochrome complex (SDHC), part of mitochondrial complex II, is a membrane anchor subunit that transports electrons from succinate to ubiquinone [49]. The model consists of four proteins: flavoprotein, iron–sulphur protein, SDHC and SDHD anchor proteins, each consisting of three transmembrane helices. The heme is bound between the SDHC and SDHD subunits. PDB ID: 1ZOY was used as template to build the hetero 4-mer complex model. It has (RMSD of 0.287 and TM-score = 1) between the model and the template and a MolProbity score of 3.13.

### Predictions of the impacts of mutations

The UniProt viewer (variants section) can show mutations not only from UniProt but also from large-scale studies, such as COSMIC and 1000-Genomes. A colour scale is used that highlights the differences between deleterious and benign mutations. A green colour indicates no associated disease, whereas a red colour indicates disease-causing mutations according to UniProt curation. Mutations highlighted in light to dark blue have been detected in large-scale studies (Supplementary Figure S3, see Supplementary Data available online at http://bib.oxfordjournals.org/). Two sequence-based methods, PolyPhen-2 and SIFT, were used by UniProt to predict the impacts of these mutations as benign or deleterious. Missense mutations reported in COSMIC CGC and highlighted in the mutation table on COSMIC CGC 3D have predicted values from structural-based methods, SDM and mCSM (and variant algorithms: mCSM-stability, mCSM-NA and mCSM-PPI). A value equal or higher than +2 indicates a mutation highly stabilizing, whereas a value equal to or lower than −2 suggests that mutation is highly destabilizing.

**Figure 4:**
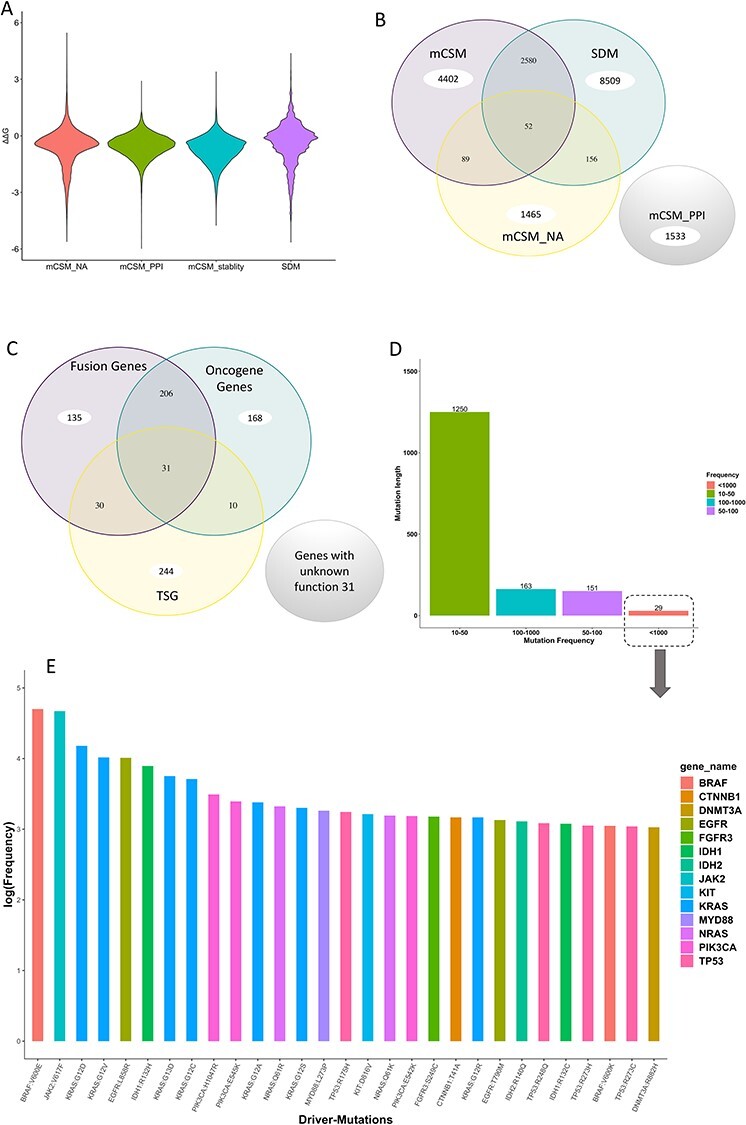
Analysis of mutations with structural annotations deposited in the COSMIC CGC 3D database. (**A**) The distribution of impacts of mutations predicted by SDM and mCSM (nucleic acid, PPI and stability). (**B**) The resemblance of highly destabilizing mutations between mCSM (stability, NA and PPI) and SDM tools. (**C**) The overlap annotations of fusion genes, oncogene genes or tumour-suppressor genes in the COSMIC CGC 3D (**D**). Frequencies of the most mutated residues reported in COSMIC CGC with a structural annotation in COSMIC CGC 3D database. (**E**) Frequencies (log) of the most frequently mutated residues, each of which has over 1000 occurrences (8 of these occur in KRAS and 4 occur in TP53). Each gene is coloured differently.

The majority of the frequent mutations reported in COSMIC CGC have structural annotations in COSMIC CGC 3D. Most frequent mutations shown occur between 10 and 50 times (Figure 4**C**). Only 29 mutant residues occurring in 14 different genes have been reported for more than 1000 times. All these mutants appear in genes that are considered as hallmarks in COSMIC CGC, with the exception of DNMT3A (Figure 4**D**). These mutations are possible drivers, as they occur at essential regions in the protein, such as the binding site in KRAS (G12D) and DNA–protein interface in DNMT3A (R882H). The most mutated gene reported in the COSMIC CGC with structural annotation in the COSMIC CGC 3D database is TP53 with 25 068 missense mutations, whereas the least mutated gene is chromosome-15 open reading frame 65 (C15ORF65) with 3 missense mutations.

Many of frequently reported mutations in the COSMIC CGC 3D have not been predicted to be highly destabilizing by mCSM stability and SDM; however, mutations may affect protein–protein, protein–nucleic acid, protein–ligand, protein–metal and other interactions. Interestingly, we have observed multiple infrequent mutations predicted to be highly destabilizing for protein stability, PPIs and protein–DNA interactions (Supplementary Figure S4, see Supplementary Data available online at http://bib.oxfordjournals.org/), perhaps confirming that driver mutations are rare or act allosterically.

Finding rare or infrequent mutations at allosteric sites remains an arduous but essential task since they can impact both tertiary and quaternary structures. From our extensive mutational analysis on the protein 3D structures, we have proposed multiple rare driver mutations affecting protein stability, protein–DNA binding and PPIs through structural-based methods that go beyond the traditional frequency and functional site analyses (Supplementary Figure S5**A**–**C**, see Supplementary Data available online at http://bib.oxfordjournals.org/).

### Data statistics

**Figure 5:**
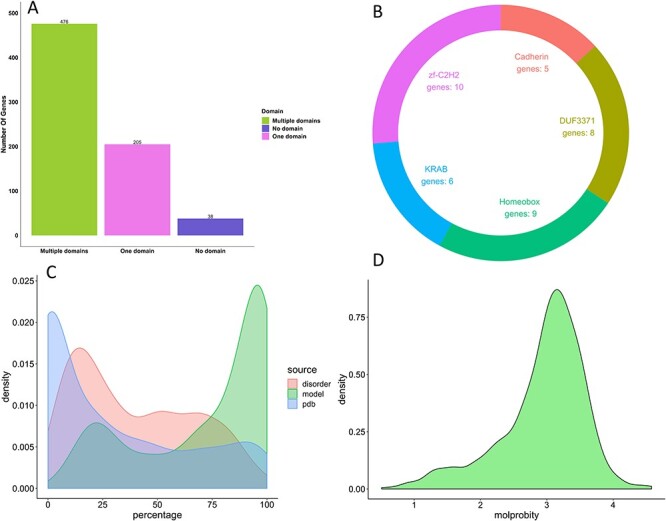
Structural analysis of genes presented in the COSMIC CGC 3D. (**A**) COSMIC CGC Pfam domain annotation; in purple, 38 genes show no domain annotations, whereas, in green and magenta, 681 genes show Pfam hits. (**B**) There are five domains annotated to be in multiple genes, of which three domains are associated with DNA binding. (**C**) Density plot for model coverage of the gene products: green colour represents modelled structures for gene products in COSMIC CGC that are close to 100% coverage of gene, whereas blue colour represents experimental structure coverage for genes in COSMIC CGC. The percentage disordered regions predicted by DISOPRED represented in red. (**D**) Density plot of the MolProbity score of all the models deposited in the CGC 3D.

There are eight genes present in the COSMIC CGC without any mutational annotations (Supplementary Table S1, see Supplementary Data available online at http://bib.oxfordjournals.org/). 283 genes in COSMIC CGC are considered as the hallmarks of cancer, as they are involved in the various metabolic pathways promoting cancer cell survival and growth. Only 59 gene products have experimental structural coverage of more than 90% of the amino acid sequence, whereas 410 gene products have structures of regions/domains that are stable and have been solved independently. There are 476 genes that have more than one Pfam domain, indicating that most of the genes in the COSMIC CGC have multidomain structures. The most frequent domains are presented in Figure 5**B**, which shows that most of the COSMIC CGC genes are associated with DNA binding.

There are 119 transmembrane protein models deposited into the COSMIC CGC 3D database. Furthermore, we have modelled 402 genes with structural coverage above 80%, approximately, 60% of the total genes in the CGC. Most genes (451 genes) have a disordered region of less than 50% of the amino acid sequence, whereas 71 genes are predicted to have disordered regions of more than 80% (Figure 5**C**). The average MolProbity score for all the models deposited in the COSMIC CGC 3D is ~3.2, with SD of 0.64, and the lowest and highest values are 0.51 and 4.58, respectively (Figure 5**D**). There are 7123 mutations predicted to be highly destabilizing according to mCSM-stability and 11 297 according to SDM. Of these, 2632 mutations are identified by both mCSM-stability and SDM to be highly destabilizing (Figure 4**B**). Furthermore, 1458 mutations are predicted to be destabilizing on the PPI and 1710 are predicted to be destabilizing at the protein–DNA interface. On the other hand, there are 34 mutations predicted to be stabilizing by mCSM Stability, 1303 mutations predicted to be stabilizing by SDM, 2 mutations predicted to be stabilizing by mCSM-PPI and 158 mutations predicted to be stabilizing by mCSM-NA.

## Discussion

In the past few years, proteomic and genomic databases, such as Genome3D, ModBase [50], COSMIC, COSMIC-3D, Pfam, GenBank [51] and UniProt, have gradually become more widespread, providing extensive information about protein sequences, functions, domain annotations and mutational data. However, these structural databases are either limited to experimental structures or to single domain modelling. Our approach is based on comparative homo- and hetero-oligomeric complex modelling to understand the impacts of mutations on protein structures and the interactions between domains, subunits and different proteins. Structures of more complex, large proteins and assemblies are increasingly becoming available by cryo-EM. However, challenges remain in characterizing single conformations and interactions in many of these, especially for those that have low complexity regions and/or that are intrinsically disordered. Nevertheless, some models of these more complex systems have been built with the approach described here, such as our recent SARS CoV-2 3D database [52], the cGMP-specific phosphodiesterase 6 (PDE6) [53].

Knowledge of protein folds, including sidechain conformations, quaternary structures and transient complexes, is essential for understanding protein stability, function and the impact of mutations. This can be achieved through the combination of experimental methods and comparative modelling, followed by energy minimization and perhaps molecular dynamics simulations, ultimately yielding more reliable *in silico* predictions of the impact of mutations using statistical or machine learning approaches.

Almost all genes in COSMIC CGC 3D feature both globular domains and intrinsically disordered protein regions (IDPRs). The IDPRs are characterized by remarkable conformational flexibility and therefore they lack stable 3D structure. The IDPRs can be located within sequences that contribute to globular structures, or in between them, before or after globular domains. Unfortunately, these regions have been usually regarded as not critical for protein function, whereas in reality, IDPRs can actually perform crucial functional roles. Additionally, multiple mutations have been observed in the IDPRs region and thus we have included the IDPR regions into our models and attempted to predict their impacts on protein stability. We believe proteins are dynamic and IDPRs deeply influence protein flexibility and oligomerization.

There remain many challenges in the modelling of the wildtype proteins. These include:

(i) The accuracy of the model depends on the sequence similarity of the modelled protein to that of the target. Where the similarity is low, loops may differ and residues may be inserted or deleted in the homologues to be modelled, leading to less accurate models.
(ii) The oligomeric state can vary between orthologues as well as paralogues. This makes automated modelling particularly challenging.
(iii) Conformations may also differ according to functional state, whether the proteins are enzymes (apoenzymes, holoenzymes or intermediate substrate-enzyme complexes), receptors or other regulatory proteins.
(iv) The majority of protein structures of the human CGC have disordered regions, often linking structured domains, which makes the assembly of the domains very challenging.

One of the advantages of our COSMIC CGC 3D database is that it takes into consideration the problems mentioned above by manually modelling the most challenging genes with high structural coverage (Figure 5**C**). We have mapped 127 443 mutations from COSMIC CGC to the modelled structures. This covers around 71% of the reported mutations in COSMIC CGC. We are continuously updating the models as new templates appear.

## Conclusion

The COSMIC CGC 3D was developed to compensate for the significant shortage of experimental 3D structures available for the most essential genes in cancer. Furthermore, the increase in the number of reported mutations leading to cancer makes it necessary to rely on protein modelling approaches, which have higher throughput. Highly intrinsically disordered regions, either inserted within the globular domains or between them, makes it very challenging to find an experimental solution quickly for the full functional complex or macromolecular assembly. Mapping the mutations to the full modelled complexes and exploiting novel methods such as SDM and mCSM will certainly increase our understanding of which mutations will likely have significant impacts on function and drug discovery and perhaps allow the design of new cancer medicines that are less susceptible to the emergence of drug resistance. The immediate future plans for the COSMIC CGC 3D database include allowing the user to build the mutant structures. We also plan to integrate further computational tools to predict the impact of mutations on protein stability, such as mCSM-lig, MAESTRO, STRUM and FOLD-X, aiming to increase the prediction accuracy and to make a consensus score for all the predicted values. Since COSMIC CGC 3D is an ongoing effort, we will update any new genes coming to the COSMIC CGC.

**Key Points**

- COSMIC CGC 3D is a comprehensive annotated database that predicted the impact of mutations on cancer gene.
- The modelled cancer proteins include homo-oligomers, hetero-oligomers, transmembrane proteins and complexes with DNA, RNA, ligands and co-factors.
- Structural-based tools, such as SDM and mCSM (protein stability, protein–protein, protein–DNA), are used to predict the impacts of mutations on the protein interfaces, protein stability and protein–DNA interface.

## Supplementary Material

Supplement-COSMIC_bbab220

## Availability

All data are freely available at https://cancer-3d.com/.

## Notes

**Ali F. Alsulami** is a PhD candidate in the Department of Biochemistry at the University of Cambridge, and he is working in the fields of cancer, SARS CoV-2 computational and experimental drug discovery.

**Pedro H. M. Torres** is a professor at the Laboratório de Modelagem e Dinâmica Molecular, Instituto de Biofísica Carlos Chagas Filho, Universidade Federal do Rio de Janeiro, Rio de Janeiro, RJ, Brasil. His research focuses on bioinformatics tools for proteomic databases and virtual screening and docking for early drug discovery.

**Ismail Moghul** is a PhD candidate at the UCL Cancer Institute, University College London. His research areas focus on bioinformatics.

**Sheikh Mohammed Arif** is a postdoc in the Department of Biochemistry, University of Cambridge. His research area focusses on *Pseudomonas aeruginosa* structure-guided fragment-based drug discovery.

**Amanda K. Chaplin** is a postdoc in the Department of Biochemistry, University of Cambridge. Her research focuses on non-homologous deoxyribonucleic acid (DNA) end joining (NHEJ).

**Sundeep Chaitanya Vedithi** is a research director of the American Leprosy Mission and leads a group of postdocs at the Department of Biochemistry, University of Cambridge, focusing on bioinformatics and drug discovery for *Mycobacterium leprae*.

**Tom L. Blundell** is a professor at the Department of Biochemistry, University of Cambridge. His research focuses on structural biology, bioinformatics and drug discovery for cancer and mycobacterial infections.
