# Supplementary material for: COSMIC Cancer Gene Census 3D database: understanding the impacts of mutations on cancer targets
Source: Brief Bioinform. 2021 Jun 17;22(6):bbab220. doi: 10.1093/bib/bbab220 (PMC8574963; doi:10.1093/bib/bbab220)
Supplement: Supplement-COSMIC_bbab220 [file supplement-cosmic_bbab220.docx]

**COSMIC Cancer Gene Census 3D database: understanding the impacts of mutations on cancer targets.**

Ali F. Alsulami, Pedro H. M Torres, Ismail Moghul, Sheikh Mohammed Arif, Amanda K. Chaplin, Sundeep Chaitanya Vedithi, and Tom L. Blundell

**Corresponding author: Tom Blundell, Department of Biochemistry, University of Cambridge, Cambridge CB2 1GA, UK. E-mail:** [**tlb20@cam.ac.uk**](mailto:tlb20@cam.ac.uk)

**Ali F Alsulami** is a Ph.D student in the department of biochemistry at university of Cambridge, working in computational and experimental drug discovery.

**Pedro Torres** is a professor at the Laboratório de Modelagem e Dinâmica Molecular, Instituto de Biofísica Carlos Chagas Filho, Universidade Federal do Rio de Janeiro, Rio de Janeiro, RJ, Brasil. His research focuses on bioinformatics tools for proteomic databases and virtual screening and docking for early drug discovery.

**Ismail Moghul** is a PhD candidate at UCL Cancer Institute, University College London. His research areas focus on bioinformatics.

**Sheikh Mohammed Arif** is a postdoc in the Department of Biochemistry, University of Cambridge. His research area focusses on pseudomonas aeruginosa structure-guided fragment-based drug discovery

**Amanda Chaplin** is a postdoc in the Department of Biochemistry, University of Cambridge. Her research focuses on Non-homologous DNA end joining (NHEJ).

**Sundeep Chaitanya Vedithi** is a research director of the American Leprosy Mission and leads a group of postdocs in the Department of Biochemistry, University of Cambridge, focusing on bioinformatics and drug discovery for Mycobacterium leprae.

**Tom Blundell** is a professor at the Department of Biochemistry, University of Cambridge. His research focuses on structural biology, bioinformatics and drug discovery for cancer and mycobacterial infections.

Supplementary Material


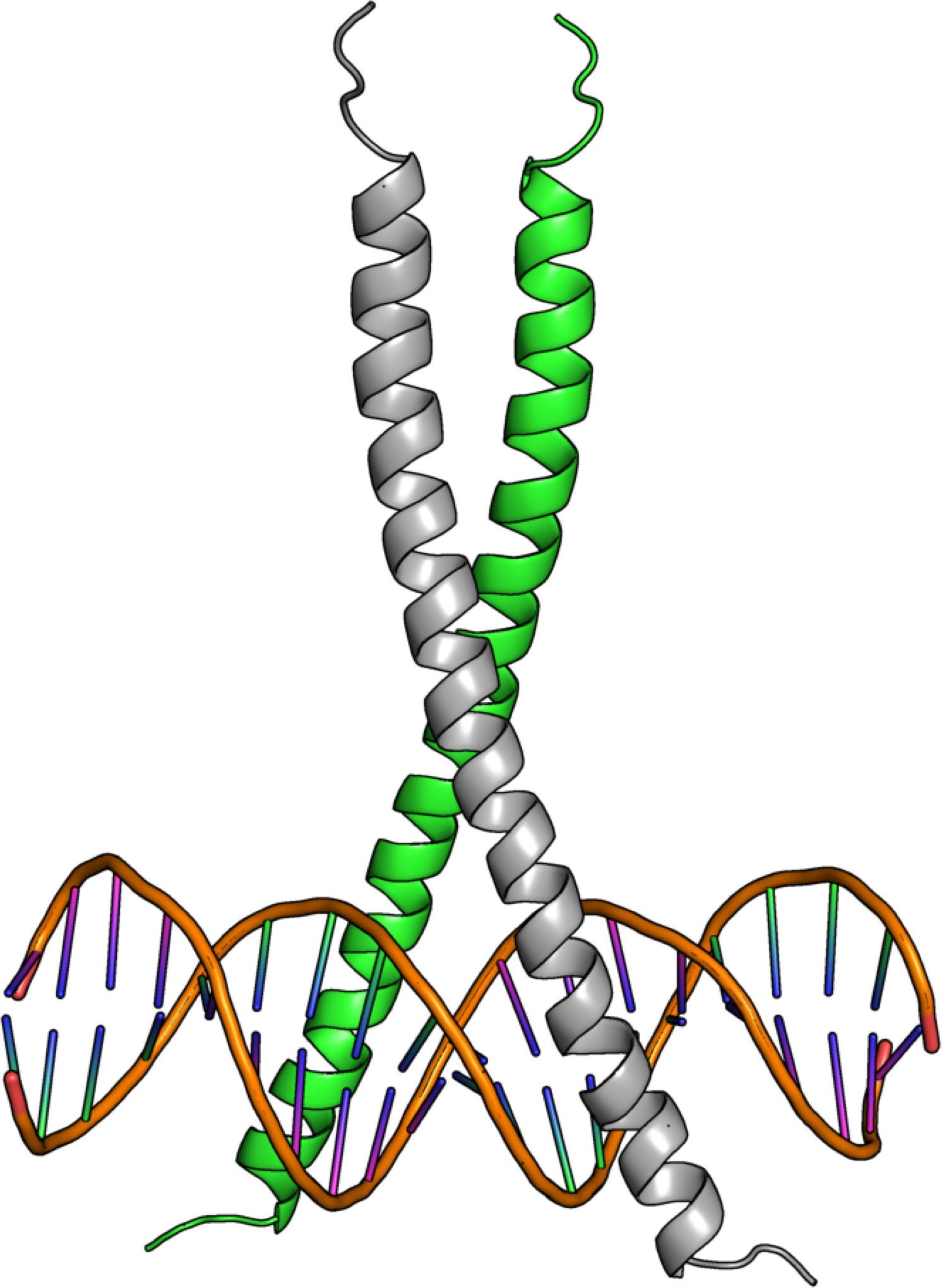


**Figure S1-** Cyclic AMP-dependent transcription factor ATF-1, modelled as homodimer, coloured in green and gray and DNA in light orange, based on (PDB ID; 5ZKO) template. The MolProbity score for this modelled structure is 2.84.


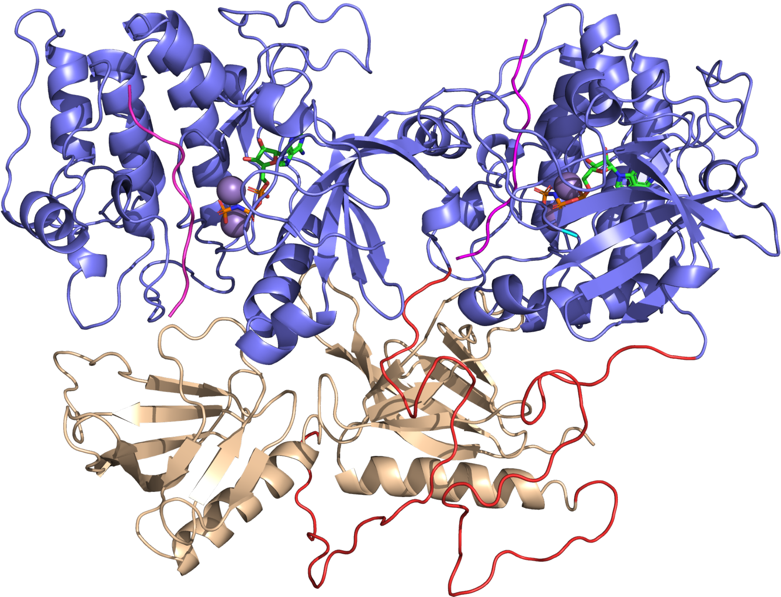


**Figure S2-** RAC-beta serine/threonine-protein kinase (AKT2) PH domain (PDB ID: 1P6S), covering the region between amino acids 1-111 coloured in wheat, intrinsically disordered region between 112-145 coloured in red, and a protein kinase domain (PDB ID:3D0E) covering the region between 146-480 coloured in light purple blue. ANP (PHOSPHOAMINOPHOSPHONIC ACID-ADENYLATE ESTER) ligands in stick, manganese ions in spheres dark purple blue, and GLYCOGEN SYNTHASE KINASE-3 BETA in magenta. The MolProbity score for this modelled structure is 2.98.


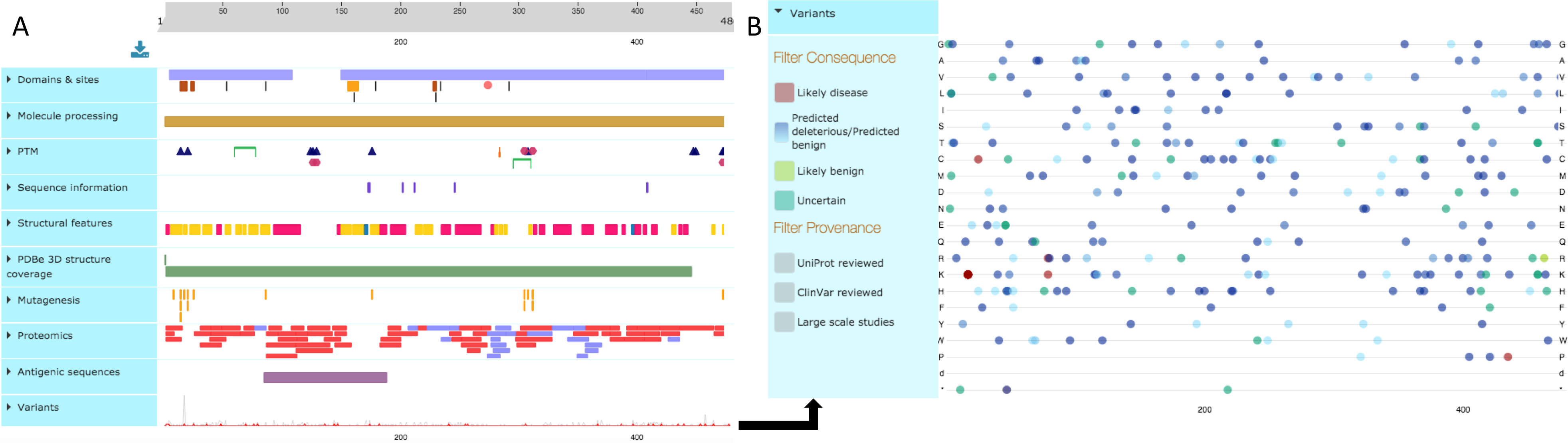


**Figure S3-** (A) UniProt viewer with sequence features of important biological data such as domains, topology, post-translational modifications, etc. (B) Variant included mutations from large-scale studies such as COSMIC, gnomAD, mutations coloured in red are likely to be associated with diseases whereas mutations coloured from light-blue slightly deleterious to dark-blue which likely to be deleterious based on computation prediction from a sequence-based method; SIFT, and PolyPhen**.**

**Table 1. Genes presented in COSMIC Cancer-Gene-Census without mutation annotation.**

| Gene Symbol | Gene ID | Gene name |
| --- | --- | --- |
| TRA | 6955 | T cell receptor alpha locus |
| TRB | 6957 | T cell receptor beta locus |
| TRD | 6964 | T cell receptor delta locus |
| IGH | 3492 | immunoglobulin heavy locus |
| IGK | 50802 | immunoglobulin kappa locus |
| IGL | 3535 | immunoglobulin lambda locus |
| HMGN2P46 |  | high mobility group nucleosomal binding domain 2 pseudogene 46 |
| MALAT1 |  | metastasis associated lung adenocarcinoma transcript 1 (lnc-RNA; non-protein coding) |

**APIs usage**

The currently implemented API includes queries by gene_id, gene_name, and uniprot_id represented on the queries section. A query takes a single identifier such as 90, or ACVR1, or Q04771, and returns the information about the gene in JSON files.

Examples

- https://cancer-3d.com/api/models/(Uniprot_id = Q04771) OR (Gene_id = 90) OR (Gene_name = ACVR1)
- https://cancer-3d.com/api/mutations/(Uniprot_id = Q04771) OR (Gene_id = 90) OR (Gene_name = ACVR1)


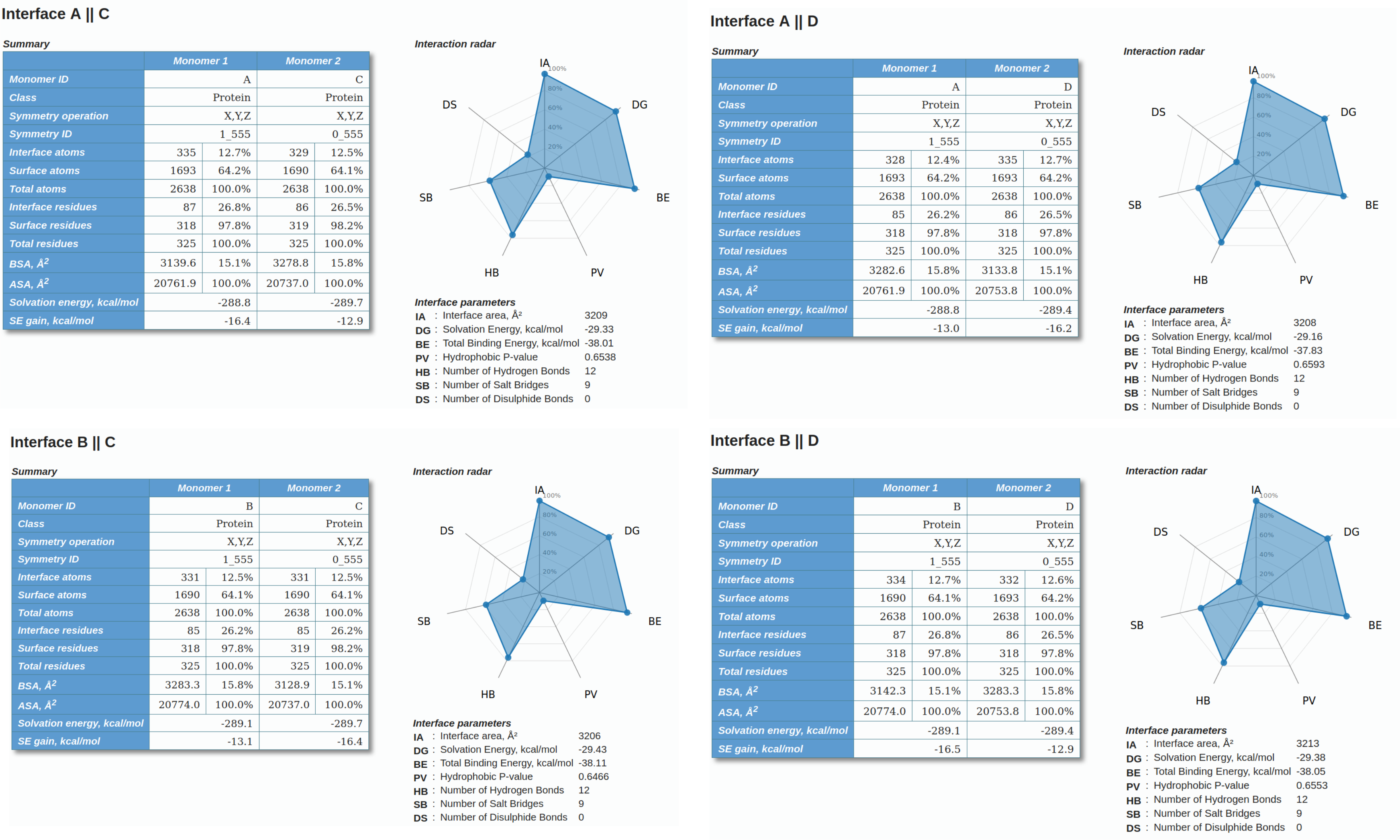


**Figure S4** Interaction radar for the G-protein-activated inward rectifier potassium channel 4 (KCNJ5) homo 4-mer interface.


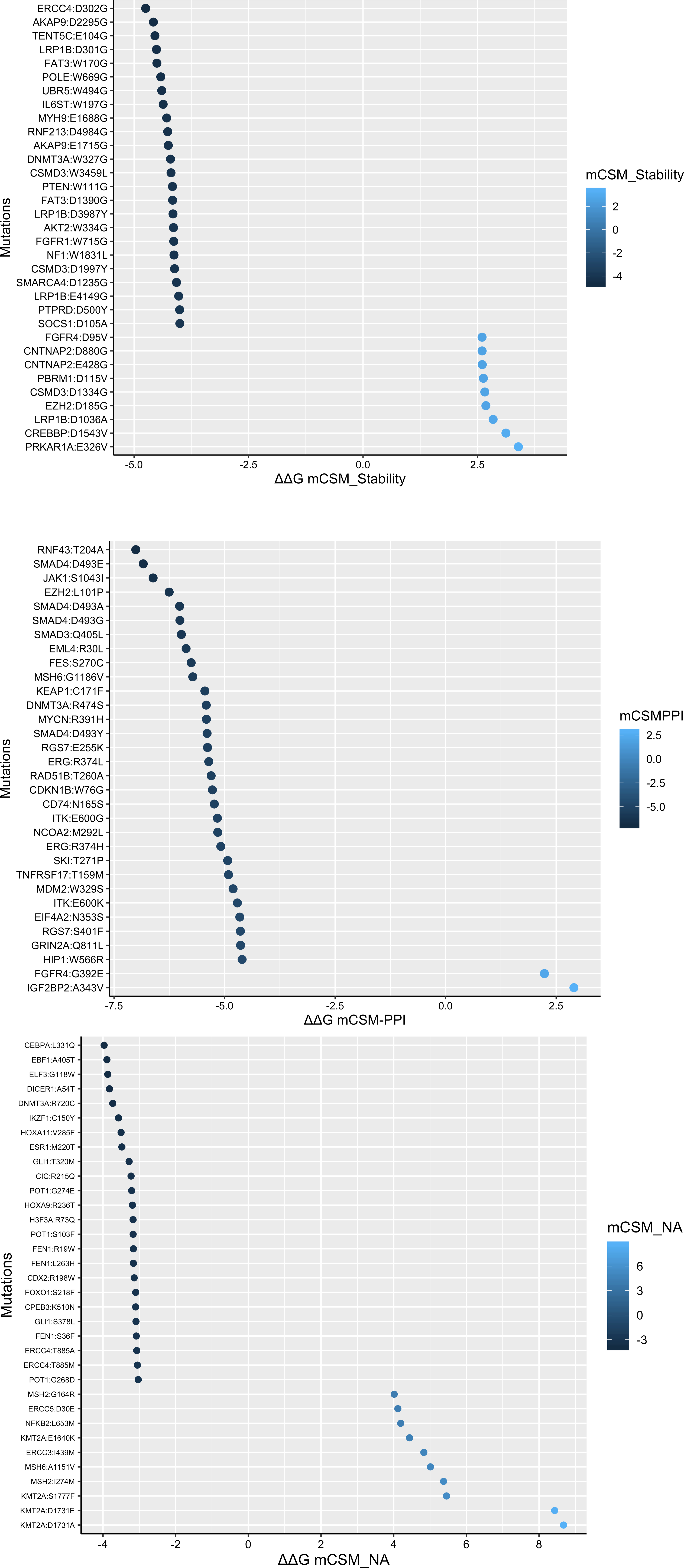


**Figure S5**. Prediction of impacts of rare mutations with a structural representation in the CGC-3D: (A) predicted highly destabilising residues in (black-to-blue) and stabilising residues in (light blue) on the protein-protein interface by mCSM-PPI. (B) Predicted highly destabilising (black-to-blue) and stabilising (light blue) residues at the DNA protein interface by mCSM-NA. (C) Amino acid residues predicted as highly destabilising by both mCSM-Stability (black-to-blue) and SDM (light blue).
